# Diagnostic and prognostic serum marker of cholangiocarcinoma (Review)

**DOI:** 10.3892/ol.2014.2696

**Published:** 2014-11-10

**Authors:** XIAOJUN ZENG, HUALIN TAO

**Affiliations:** 1 Department of Laboratory Medicine, Luzhou Medical College, Luzhou, Sichuan 646000, P.R. China; 2 Department of Laboratory Medicine, Affiliated Hospital of Luzhou Medical College, Luzhou, Sichuan 646000, P.R. China

**Keywords:** cholangiocarcinoma, serum marker, diagnostic, prognostic

## Abstract

Cholangiocarcinoma (CCA) is a fatal disease that is typically diagnosed late and treated ineffectively. As the morbidity and mortality rates for CCA rise markedly, patietns with CCA currently have a poor prognosis. However, if it were possible to diagnose CCA early while effective treat methods are available, CCA patients would achieve a better quality of life. Therefore, preventing the process of CCA in the early stages is an urgent problem to solve. An accurate, quick and safe method to diagnose early-stage CCA is required. The present review discusses the risk factors, status of research and certain serum markers of CCA. The sensitivity and specificity of these markers differ from each other. To explore the more accurate serum markers may be a novel direction and method for the diagnosis of CCA in laboratory medicine in the future.

## 1. Introduction

At present, the term cholangiocarcinoma (CCA) includes all bile duct cancers, consisting of 20% intrahepatic, ~55% perihilar, a subset of extrahepatic CCAs which involves the bifurcation of the ducts, 20% distal extrahepatic CCA, and the remaining 5% of CCAs are multifocal (1). CCA is an aggressive malignancy that, due to early invasion, widespread metastasis and the lack of an effective therapy, is associated with a high incidence and mortality rate (2). While CCA is relatively rare, the incidence rates have increased markedly worldwide in the past decade (3). In Thailand, particularly in the Northeastern region, CCA is a crucial health problem that is caused by a combination of *Opisthorchis viverrini* (OV) infection and nitrosamine (4). This trend in the incidence of CCA is followed by other regions of Southeast Asia and China (5). In England and Wales, from 1997–1998, CCA caused almost 1,000 mortalities per year (6).

## 2. Risk factors

In intrahepatic cholangiocarcinoma (ICC), several risk factors have been established, including primary sclerosing cholangitis (PSC), fibropolycystic liver disease, parasitic infection, intrahepatic biliary stones and chemical carcinogen exposure. Tanaka *et al* (7) performed a large-scale cohort study in the province of Osaka (Japan) and found that hepatitis B virus (HBV) infection and liver inflammation are independently associated with ICC development, even though there remains a requirement for further large cohort studies to verify these findings. A meta-analysis by Li *et al* revealed that HBV is associated with an increased risk of CCA, particularly for ICC (8). As the incidence of ICC is rising with the emergence of hepatitis C virus (HCV), Sempoux *et al* (9) considered that HCV infection is also a risk factor.

In perihilar cholangiocarcinoma, a variety of risk factors have been identified, including advanced age, male gender, PSC, choledochal cysts, cholelithiasis, cholecystitis, parasitic infection, including with OV and *Clonorchis sinensis*, inflammatory bowel disease, alcoholic and nonalcoholic cirrhosis, chronic pancreatitis and metabolic syndrome (10).

For extrahepatic cholangiocarcinoma (ECC), studies reporting the risk factors appear to be fewer compared with those for ICC and perihilar CCA. However, it is indicated that blood type A is more common in younger ECC patients, and there is a lower proportion of serum carbohydrate antigen (CA)-125 and elevation of CA19-9 in patients with blood type A. Blood type A and HBV infection are associated with ECC risk, and there is a synergism of blood type A and HBV infection in the development of ECC (11).

## 3. Status of research

As the incidence of CCA is rapidly increasing, numerous investigations focus on this disease. According to experimental evidence, CCA can be classified into three types, CCA cell lines, animal models, and human tissue, blood and bodily fluid. Currently, a large amount of CCA data, covering all fields of this disease, has been published and involves studies into genes (12–14), proteins (14), metabolic enzymes (15), signaling pathways (13) and specific cells (16–19).

## 4. Serum makers

CCA is associated with a late presentation and difficult diagnosis (20). At present, a confirmed diagnosis of CCA continues to rely on histopathological examination, an invasive method, despite the sample being difficult to obtain. The sample for another important examination, cytology, is also difficult to obtain and these methods possess the same risk of seeding (21). This emphasizes the need for serum tumor markers that are safe and exhibit high specificity and sensitivity.

### CA19-9

CA19-9 is a serum tumor marker for pancreatic cancer, CCA and other malignancies with a suboptimal sensitivity and specificity. Li *et al* (22) found that the sensitivity and specificity of CA19-9 are 68.4 and 75%, respectively. Leelawat *et al* considered the sensitivity and specificity of serum CA19-9 as a serum marker, with a cut-off value of 100 units/ml, to be 68 and 87%, respectively. In conclusion, the accuracy of CA19-9 in identifying CCA is not high (23).

Singh *et al* (24) also found that in CCA patients, CA19-9 exhibited poor clinical utility as a tumor marker and did not alter patient management. The elevation in CA19-9 was suggested by the results to be associated with biliary obstruction based on clinical history, laboratory data and diagnoses. In addition, the elevated levels of serum CA19-9 and incomplete removal of stones were potential predictive factors for CCA in patients with hepatolithiasis (25). These results may indicate that CA19-9 is associated with biliary obstruction, but a large-scale clinic investigation is required to verify the findings.

Although CA19-9 serves as a serum maker for CCA, it is not satisfactory and there may be certain factors affecting the value. For example, allelic variants of fucosyltransferase (FUT)2 and 3 affect the serum levels of CA19-9. In PSC patients, FUT2 and 3 levels are a screening parameter commonly used to evaluate the biliary malignancy. Based on the different genotypes of FUT3 and FUT2, which can determine the cut-off level of CA19-9, and the level of CA19-9 in 433 PSC patients, 41 of whom possessed biliary malignancy, Wannhoff *et al* (26) classified the PSC patients into three groups (no FUT3 activity regardless of FUT2 activity, both FUT2 and FUT3 activity, and no FUT2 activity without loss of FUT3 activity, respectively). Youden’s index and receiver operating characteristic curve (ROC curve) revealed that the exclusive cut-off values for each group could increase the sensitivity to 90% and reduce the false positive results. Previously, Sinakos *et al* (27) also found that numerous patients with PSC and increased serum levels of CA19-9 did not suffer from CCA. In conclusion, when determining the level of CA19-9 in PSC patients, the cut-off value should be modulated based on FUT2/3. Therefore, additional studies are required to explore whether there are other factors affecting CA19-9 levels.

CA19-9 is not only used for diagnosis, but also for prediction. A meta-analysis was performed to determine the prognostic role of pre-operative serum CA19-9 levels in the survival of patients with CCA. The results revealed that a pre-operative elevation in the CA19-9 levels of CCA patients was correlated with a poor prognosis (28).

In hilar CCA, a subgroup of CCA, the combination of CA19-9 and carcinoembryonic antigen (CEA) serum levels are associated with tumor stage. The high pre-operative serum levels of CA19-9 and CEA in hilar CCA patients demonstrated a decreased survival time and an increased incidence of irresectability (29).

### S121 or CCA-CA and CA-S27

Silsirivanit *et al* (30) established a novel monoclonal antibody (MoAb) using pooled CCA tissue, they obtained an S121 immunoglobulin M MoAb that recognized a novel glycan epitope. Their findings demonstrated that mucin 5AC (MUC5AC) is a core glycoprotein for the S121 epitope. The serum S121 level was able to differentiate CCA patients from healthy individuals, active OV-infected individuals and patients with various gastrointestinal cancers, hepatoma and benign hepatobiliary diseases with high sensitivity (87.63%), specificity (89.58%), positive predictive value (80.95%) and negative predictive value (93.47%). High serum S121 levels were also associated with a poor patient outcome. In addition, the results from a golden hamster model of CCA suggested that CCA-CA is a potential marker for the early diagnosis of CCA (31).

CA-S27, is a novel Lewis a (Lea)-associated carbohydrate epitope and modification of MUC5AC mucin. A high level of CA-S27 was detected in the serum of CCA patients and was able to differentiate CCA patients from patients with gastrointestinal cancers, hepatomas, benign hepatobiliary diseases and healthy subjects, exhibiting a high sensitivity (87.5%) and high negative predictive value (90.4%). Also, patients with high serum CA-S27 exhibited a significantly shorter survival time compared with those possessing a low serum CA-S27 level, regardless of the serum MUC5AC levels. Notably, FUT3 was also revealed to be a regulator of CA-S27 expression. CA-S27 is a potential diagnostic and prognostic marker in CCA (32).

### CA242

CA242 is a sialiated carbohydrate antigen that has been used as a tumor marker in several tumors. The combination of α-fetoprotein(−) and CA242(+) afforded a high specificity (94.3%) and a high accuracy (78.5%) when distinguishing ICC from HCC (hepatocellular carcinoma) (33).

### Matrix metalloproteinase (MMP)-7

Serum levels of MMP-7 were significantly elevated in CCA patients (P<0.001), which indicates that MMP-7 is a useful biomarker in differentiating CCA from benign biliary tract obstructive diseases (23). The sensitivity and specificity of serum MMP-7 is 53–76.32% and 46.88–92%, respectively, but at different cut-off values, this varies (Table I). ROC curve analysis revealed that the detection of the serum MMP-7 level is a reasonably accurate method for the differentiation of CCA from benign biliary tract disease patients [area under curve (AUC), 0.73–0.84] (23,34,35).

The ROC curve analysis of another study revealed that the MMP-7 level could distinguish between CCA and other hepatobiliary diseases, including opisthorchiasis, benign biliary disease, HCC, hemangioma (P<0.001; AUC, 0.775) (35). However, in order to confirm whether MMP-7 could distinguish CCA from other cancers or biliary tract diseases, a large-cohort study would be required. Additionally, the process of setting up an accurate cut-off value for MMP-7 is also an issue.

### Apolipoprotein (Apo)A-I and neutrophil gelatinase-associated lipocalin (NGAL)

ApoA-I is a member of apolipoprotein A1/A4/E family that is synthesized in the liver and small intestine (36). Downregulated ApoA-I in CCA patients may be linked to liver and small intestine dysfunction. ApoA-I may differentiate CCA from benign diseases of hepatobiliary and healthy individuals (AUC, 0.883) and other cancers including laryngopharyngeal, laryngeal, esophageal, prostate and lung cancer (AUC, 0.715). Therefore, it is suggested that ApoA-I could be a potential useful biomarker for CCA (37).

NGAL is a 25-kDa glycoprotein that belongs to the lipocalin superfamily (38) and regulates the action of gelatinase (39). Srisomsap *et al* (40) demonstrated that NGAL is a secreted protein that is only expressed in the conditioned media of the CCA cell line. Investigating NGAL expression in various types of cancer and normal tissues obtained from CCA patients revealed that NGAL was expressed only in the cancer tissues. High NGAL expression has been reported in various cancers and, among these cancers, the NGAL level was significantly elevated in CCA patients (P<0.001). The AUC of the ROC curve was 0.79. NGAL is promising to serve as a biomarker to discriminate CCA patients from benign biliary tract disease patients (38).

### α1-antitrypsin (A1AT) and oxidized (ox)-A1AT

A1AT is a 52-kDa glycoprotein produced by hepatocytes and mononuclear phagocytes, and is the principal human inhibitor of neutrophil elastase (NE) (41). A1AT was found to be expressed in the tumors of CCA patients (42). In CCA patients, the plasma level of A1AT is upregulated and is significantly higher compared with control subjects. Serum levels of CA19-9 combined with A1AT were the minimum requirement to obtain a predictive accuracy of >80% in diagnosing CCA (43).

Ox-A1AT is a modified form of A1AT, which is generated by free radicals, released from inflammatory cells, oxidising the active site of A1AT. In patients with heavy OV infection, advanced periductal fibrosis (APF) and CCA, the serum levels of ox-A1AT were significantly higher compared with healthy controls (P<0.001). Ox-A1AT can therefore serve as a potential risk indicator for *Opisthorchiasis*-associated CCA (42).

### KL-6 mucin

KL-6 mucin is a type of MUC1 mucin that may play an important role in unfavorable tumor behaviors, including the invasion and metastasis of carcinoma of the ampulla of Vater (44). In liver cancer tissues, KL-6 mucin is only expressed in ICC (45).

The serum KL-6 mucin level was elevated in ICC patients. When the cut-off value is set at 248 units/ml, the positive rate of ICC patients, healthy individuals and HCC patients is 100.0, 15.8 and 18.5%, respectively. KL-6 is a potential serum maker for differentiating ICC from HCC (45).

### Exostosin (EXT) 1

EXT1 is a novel biomarker that has been studied in CCA. Khoontawad *et al* (46) screened markers in the plasma of hamsters that were infected with OV and treated with N-nitrosodimethylamine (NDMA) to induce CCA. The most frequently upregulated protein, EXT1, was validated by western blot analysis. In human CCA, the plasma EXT1 level was significantly higher compared with healthy controls. The results of this study indicated that increased expression of EXT1 in the plasma may be involved in CCA genesis and be a potential biomarker of CCA.

### α1β-Glycoprotein (A1BG)/afamin (AFM) ratio

A1BG was identified and confirmed as an elevated protein in pancreatic juice and cancer tissues of pancreatic ductal adenocarcinoma. A1BG may be a cancer-associated gene and a novel tumor marker for cancer (47). AFM, a member of the albumin family, is a vitamin E-binding protein (48) that was reported as a novel protein marker for ovarian cancer (49) and may be a negative acute-phase protein, although the pathophysiology is unclear at present (50).

A1BG and AFM were detected consistently at different degrees in CCA serum compared with controls. Tolek *et al* (51) selected the two types of protein using proteomes. The serum A1BG/AFM ratio has a high specificity (87.5%) and sensitivity (84.4%) in diagnosing CCA, as the ratio is significantly higher in CCA compared with the control.

### Orosomucoid (ORM) 2 and kinesin (Kif) 18A

ORM2 is a key acute-phase plasma protein in inflammation and is reported to be a novel member of cancer-associated glycoproteins (52). Kif18A is a microtubule depolymerase, a key regulator of chromosome congregation and a member of the kinesin superfamily of molecular motor proteins (53). In hamster CCA models induced by NDMA, OV infection and OV + NDMA (ON), respectively, Rucksaken *et al* (54) found that the plasma level of ORM2 was overexpressed in the OV and ON groups and Kif18A was overexpressed in the ON group at the precancerous stage, which was verified by western blotting. In conclusion, these results suggested that Orm2 and KIF18A may serve as potential biomarkers for early diagnosis of CCA.

### Adhesion molecule (ICAM)-1

ICAM-1 is a member of the immunoglobulin superfamily of adhesion molecules and was reported to be associated with melanoma (55). ICAM-1 is important in cell-cell and cell-basement membrane interactions (56) and, in normal vascular endothelial cells and mononuclear macrophages located in the small intestine and lymph, ICAM-1 is expressed at low levels (57).

In CCA, the serum ICAM-1 level is significantly higher compared with healthy controls and exhibits high sensitivity (77%) and specificity (70%) at a cut-off value of 167 ng/ml. Positive correlations between ICAM-1 and alkaline phosphatase and CEA levels were also identified. However, there was no significant difference between the serum ICAM-1 levels in CCA and benign biliary diseases with mainly inflammatory features. As the serum level of ICAM-1 significantly decreased following tumor resection, it may serve as a prognostic maker in CCA (58).

### Pyruvate kinase isoenzyme type M2 (M2-PK)

M2-PK is a novel tumor metabolic marker (59) that plays a key role in tumor metabolism and energy production by channeling glucose carbons, either into synthetic processes or toward glycolytic energy production. Increased aerobic glycolysis is one of the most common metabolic phenomenons in tumor cells, which particularly express M2-PK (60). As a result, aerobic glycolysis causes the accumulation of glycolytic phosphometabolites, allowing cells to invade areas with low concentrations of oxygen and glucose (61).

In CCA patients, the level of serum M2-PK was markedly higher compared with controls (P<0.05) and exhibited a high sensitivity (84.2%) and specificity (90%). M2-PK is maybe a promising maker in diagnosing CCA (22).

### Hydroxyproline (HYP) and collagen I

HYP is an unique amino acid for maintaining cell structure and function, and is a major component of collagen (62). Excessive acids, MMPs and cathepsin K, could degrade collagen I into HYP (63). Prakobwong *et al* (35) found that the plasma levels of HYP and collagen I were significantly higher in opisthorchiasis patients compared with healthy individuals. The highest plasma level of HYP in the hepatobiliary disease patients was observed in CCA patients (P<0.05). The HYP and collagen I levels significantly differed between *Opisthorchiasis*, benign biliary disease and CCA patients (P<0.001). Notably, only the plasma HYP level in benign biliary disease was predictive of CCA risk (OR, 3.69; P=0.020) (35).

### Golgi membrane protein (GOLM) 1

GOLM1 is transmembrane protein that is expressed in the Golgi of epithelial cells. Previous studies have indicated that the expression of GOLM1 is upregulated in acute and chronic liver diseases (64) and is useful in the diagnosis and therapy of prostate cancer (65). The overexpression of GOLM1 is associated with poor prognosis in human HCC (66). Kristiansen *et al* (64) used a membrane protein enrichment strategy in addition to ^18^O labeling-based quantitative proteomics to identify proteins that are highly expressed in CCA. This study confirmed GOLM1, Annexin IV and epidermal growth factor receptor pathway substrate (Eps8) to be candidate biomarkers for CCA. Among these candidates, GOLM1 is detectable in serum and it is potentially able to serve as a biomarker for the early detection of CCA.

### Angiotensin-converting enzyme (ACE)

ACE is an important molecule in the renin-angiotensin system (RAS) (67) and also plays an important physiological role in regulating blood pressure (68). Beyazit *et al* (67) found that serum ACE levels were significantly higher in patients with ECC (56.6±27.4 units/l) compared with choledocolithiasis patients (32.9±14.6 units/l) and control groups (28.6±10.6 units/l). Circulating ACE in the RAS may be associated with ECC development through the formation of a local environment that is enriched with cytokines and other growth factors, possibly promoting cholangiocyte turnover.

### Vitamin D3

The effects of vitamin D3 have been investigated in various tumors (69) and vitamin (1,25(OH)2D) exhibits anti-proliferative effects in several cancers (70). Kennedy *et al* (70) found the expression of the vitaminD3 receptor and cytochrome P450, family 24, subfamily A, polypeptide 1, an enzyme that degrades vitamin D3, increased in CCA lines and biopsy samples compared with controls. The expression of cytochrome P450, family 27, subfamily B, polypeptide 1, an enzyme that synthesizes vitamin D3 and signals via the nuclear vitamin D3 receptor, was decreased or unchanged. In this study, it was suggested that modulation of vitamin D3 synthesis may be important in the management of CCA. As a result, the possibility of a change in serum level of vitamin D3 has been considered, and monitoring the level is a probable method for managing CCA. A large number of studies is required to explore this hypothesis.

### Estrogen

Estrogenic response is known to be a promoting factor in the progression of certain cancers. Hunsawong *et al* (71) identified a significant increase in the serum estrogen level of male CCA patients. The levels were positively correlated with serum bilirubin and alkaline phosphatase, and negatively correlated with albumin. Estrogen also demonstrated an association with the survival times of patients.

### γ-Glutamyltransferase (GGT)

GGT is one of the enzymes used for the evaluation of liver function. Yin *et al* (72) found that GGT level was an independent predictor of tumor recurrence in ICC patients. Elevation of serum GGT levels is an indicator of aggressive tumor behavior and a predictor of poor clinical outcomes.

### C-reactive protein (CRP) and albumin

CRP and albumin are commonly tested for in serum biochemical examinations. The Glasgow prognostic score (GPS), an inflammation-based prognostic score (73), is useful in predicting the outcome for a variety of cancers. In ECC patients, >10 mg/l CRP and <35 g/l hypoalbuminemia are allocated a score of 2, and one or none of these abnormalities is allocated a score of 1 or 0, respectively. As a result, increased GPS was found to be an independent predictive factor with poor prognosis in ECC by a Cox model (74).

## 5. Summary

Local invasiveness and regional lymph node and distant organ metastasis is accountable for CCA morbidity and mortality (75). CCA is often diagnosed at an advanced stage (76). As numerous useful examination methods are invasive and dangerous, safer methods are required. Currently, there are no satisfactory serum makers that can accurately differentiate CCA from other diseases. The present review discussed certain traditional and novel serum makers that could aid in the diagnosis of CCA and in determining the prognosis of CCA, but the sensitivity and specificity of these markers are far from the present expectations. In future studies, it is necessary for additional useful serum makers to be identified.

Currently, there are certain methods for investigating novel serum markers, including proteomics, lipidomics, glycomics and microRNA (mi-RNA). As carcinogenesis possesses a complex and long progression, it will exhibit a series of serum characteristics in the process. Searching for markers using animal models that mimic the development of the disease is, perhaps, an indispensable step and, during the progression of the disease, observing the changes in the serum is also essential.

## Figures and Tables

**Table I:** Sensitivity and specificity of serum MMP-7 at different cut-off values in distinguishing CCA from benign biliary tract disease.

Cut-off value, ng/ml	Sensitivity, %	Specificity, %
5.5^a^	75	78
6.0^b^	76.32	46.88
6.5^a^	63	87
7.4^b^	63.16	71.88
7.5^a^	53	92
17.0^c^	75.8	72.5
23.0^c^	55.9	83.5

a 59 CCA patients and 128 benign biliary tract diseases patients (23);

b 44 CCA patients and 36 benign biliary tract diseases patients (34);

c 37 CCA patients and 31 benign biliary disease patients (35).

CCA, cholangiocarcinoma; MMP-7, matrix metalloproteinase-7.
